# Associations of combining paid work and family care with gender-specific differences in depressive symptoms among older workers and the role of work characteristics

**DOI:** 10.5271/sjweh.4014

**Published:** 2022-03-31

**Authors:** Femmy M Bijnsdorp, Allard J van der Beek, Marjolein I Broese van Groenou, Karin I Proper, Swenneke G van den Heuvel, Cécile RL Boot

**Affiliations:** 1 Department of Public and Occupational Health, Amsterdam UMC, Vrije Universiteit Amsterdam, Amsterdam Public Health research institute, Amsterdam, The Netherlands; 2 Department of Sociology, Vrije Universiteit Amsterdam, Amsterdam, The Netherlands; 3 Centre for Nutrition, Prevention and Health Services, National Institute for Public Health and the Environment, Bilthoven, The Netherlands; 4 Netherlands Organization for Applied Scientific Research, TNO, Leiden, The Netherlands

**Keywords:** employment, gender, mental health, The Netherlands

## Abstract

**Objectives:**

This study aims to provide insight into (i) how the combination of paid work and family care is longitudinally associated with gender-related differences in depressive symptoms and (ii) the role of work characteristics in this association.

**Methods:**

Data were derived from STREAM, a Dutch prospective cohort study of older workers aged 45–64 years. Respondents were included if they were employed in at least one measurement between 2015 and 2017 (N=12 447). Mixed-models were applied to disentangle between-person (BP) and within-person (WP) effects of family caregiving on depressive symptoms. Analyses were stratified by gender. Work characteristics (social support, autonomy, emotional and mental workload) were separately added to the multivariable models.

**Results:**

For older employees, family caregiving was positively associated with depressive symptoms between and within persons for both women [BP B=0.80, 95% confidence interval (CI) 0.52–1.08; WP B=0.32, 95% CI 0.08–0.56] and men (BP B=0.75, 95% CI 0.45–1.05; WP B=0.25, 95% CI 0.01–0.48). Social support at work reduced the adverse effect of family care on depressive symptoms for women (BP) and men (BP and WP). Emotional workload partly explained the effect of family care for both women and men (BP).

**Conclusions:**

The longitudinal association between family care and mental health was similar for male and female employees. Resources at work (ie, social support) could protect caregiving employees against depressive symptoms. More research is needed regarding the relative impact of the care context compared to the work context of working family caregivers.

Due to population ageing and cutbacks in residential care, there is an increased demand for family caregivers, resulting in that more people will need to combine paid work and family care at a certain point in their working career. This combination occurs in particular during midlife, when older parents increasingly need care or when a partner becomes ill at a relatively young age. Concomitantly, older workers experience a societal push to prolong their work participation until higher age by the abolishment of early retirement schemes and an increased statutory retirement age. Since health around retirement is an important predictor of healthy ageing (1), reaching the retirement age in good health is important. However, providing family care has been associated with stress, depression, and sick leave (2, 3). Research has shown that working family caregivers experienced more mental fatigue than non-caregiving workers (4, 5). About one in five working family caregivers experienced a heavy burden, and about one in seven workers who had started with family care reported deteriorated health (4). Hence, the increasing demand to combine paid work with family care could negatively impact the mental health of older workers.

In addition, research has shown that the association between mental health and work participation, family care and the combination of work and family caregiving is different for women and men (6–8). Pressure related to combining roles has been linked to an increased risk of burn-out in women (9), and working women were at greater risk of emotional difficulties by caregiving than working men (10). A meta-analysis has shown that family caregiving increased gender differences in depression and physical health, because women experienced more care-related stressors (6). Overall, women and men have different social roles, which may contribute to differences in mental health and differences in how family caregiving impacts mental health over time. Even in today’s society, gender roles predict that women generally feel more responsible for significant others, and are more willing to provide intensive family care compared to men (2, 11). Moreover, they are more often involved in family care in combination with paid work than men (2, 12).

There are also gender differences in work that could impact the combination of paid work and family care. Since women generally work fewer hours than men (2, 11), they might have more time to fulfil caregiving tasks. However, women more often work in jobs with lower autonomy and in sectors with high mental or emotional workload (eg, education or healthcare) compared to men (13, 14). Poor work characteristics may increase the challenge to combine paid work and family care as it may be more difficult to combine work schedules with caregiving. As a result, stress from work and caregiving may build up quickly. Contrarily, having a job with high autonomy might make it easier to reconcile care and work demands, which might protect against mental health deterioration. Thus, women might be at greater risk of mental health deterioration when providing family care if their workplace is not conducive to it. Further, social support at work may also play a role in the association between family care and mental health. Research has shown that having little social support contributed stronger to mental fatigue for working family caregivers than non-caregiving workers (4). Receiving social support at work is an important predictor of mental health among workers (15) and might protect against the potential negative impact of family caregiving. Prior research has indicated that social support from supervisors had a larger effect on reducing burn-out for women compared to men (16) In this sense, women might obtain more benefits from social support at work to reduce mental health issues by the combination of family care and work than men.

Gaining insight into the complex role of gender related to the effects of combining paid work and family care on health will increase the understanding of gender-related health inequalities. A lot of studies focus on associations between a combination of paid work and family caregiving and health, but many of these are based on cross-sectional data. Little is known about whether and how changes in mental health following the combination of family caregiving and work influence gender differences in mental health over time. Also, the role of work characteristics has received limited attention in former studies, while poor work characteristics (eg, low autonomy or no social support) could impact the ability to combine work and family care, and could lead to poorer mental health.

In addition, former longitudinal studies did not distinguish between inter-personal differences (context factors) and within-individual changes, while the impact of family caregiving on mental health might differ in direction or magnitude between- and within-persons for both genders. To fill this research gap, the current study used a mixed-model approach and disentangled between-person effects (the effect of caregiving versus non-caregiving on mental health) and within-person effects (the effect of starting/stopping with caregiving on mental health) for women and men separately (17). Moreover, the current study explored the role of work characteristics in both the between- and within-person associations between family care and mental health. This study aims to answer the following research questions: (i) Does the longitudinal association between family care and mental health differ between older male and female workers? (ii) What is the role of work characteristics in the longitudinal association between family care and mental health among older workers?

## Methods

### Study design and sample

Data were derived from the Study on Transitions in Employment, Ability and Motivation (STREAM). STREAM is a prospective cohort study that includes workers in The Netherlands aged ≥45 years and aims to identify under which circumstances persons participate in employment while maintaining in good health (18). All persons included in STREAM participated in the Intomart GfK Online Panel. At baseline (2010) 15 118 persons participated in the online questionnaire (response 71.5%). The study population was stratified according to 5-year age groups and employment status (eg, employed, self-employed, and non-employed). The data collection of STREAM started in October 2010 with yearly follow-up measurements onwards (with exception of 2014 and 2018). In 2015, a new sample was included in STREAM to refill the 45–49 years category (N=6752, response 62%) (19). Also, new respondents were added to the age groups 50–54, 55–59 and 60–64 years to ensure sufficient numbers in these groups.

In the current study, respondents were selected if they were employed for ≥1 hour per week in ≥1 measurement between 2015 and 2017 (see supplementary material www.sjweh.fi/article/4014, table S1). Measurements were only included when someone was an employee in that year. The final sample included 12 447 employees aged ≥45 years: employees with family caregiving tasks (2015 N=2730; 2016 N=2374; 2017 N=2171; 7275 observations) and employees without family caregiving tasks (2015 N=8484; 2016 N=6698; 2017 N=6014; 28 196 observations).

### Measurements

*Depressive symptoms*. Mental health was the primary outcome and was assessed by the presence of depressive symptoms. Depressive symptoms were defined as the presence of the symptoms dreariness, dejection, fatigue and low self-appreciation (20) and was measured using the short form of the Center for Epidemiological Studies Depression (CES-D) (21, 22). The short form CES-D consists of 10 items covering depressive symptoms experienced in the past week, by statements such as “During the past week I was bothered by things that usually don’t bother me” (22). Respondents could answer on a 4-point scale how often they experienced depressive symptoms (0=rarely or never to 3=mostly or always). The total score of the 10 items ranged from 0–30, with a higher score indicating more depressive symptoms (Cronbach’s α=0.84).

*Family caregiving*. Respondents were labeled as *family caregivers* if they had spent time on family caregiving in the past 12 months (yes/no). Family caregiving was defined in the questionnaire as “providing unpaid care for a person in the close environment, excluding care for healthy children”. This could include family members, as well as friends or neighbors (23), but we prefer to use the term family caregiver throughout the manuscript as this covers the large majority of caregivers.

*Work characteristics*. Work characteristics included hours of paid work per week (including overtime), autonomy, social support, emotional and mental workload. *Autonomy* was measured using the Job Content Questionnaire (JCQ) (24, 25) and consisted of five items, such as “Are you able to make decisions about your work on your own?”. *Social support at work from supervisor and colleagues* was based on the Copenhagen Psychosocial Questionnaire (COPSOQ) (26) and included two items regarding supervisors “How often is your supervisor willing to listen to your work-related problems?” and “How often do you receive help and support from your supervisor?”, and two items regarding colleagues (same questions with ‘supervisor’ replaced by ‘colleagues’). *Emotional workload* was measured using the COPSOQ (26) including three items, such as “Is your job emotionally demanding?”. *Mental workload* was based on the Netherlands Working Conditions Survey (NWCS) (27) and included three items such as “Does your work require a lot of your attention?”. The items regarding autonomy, social support and workload could be answered on a 5-point scale ranging from 1=always to 5=(almost) never. The answers were reversed and a mean score was calculated per subscale. A higher score indicated more autonomy, social support, emotional and mental workload.

*Socio-demographics*. Characteristics of the respondents included gender, age (in years) and partner status (yes/no). The highest level of education completed at baseline was categorized into low (pre-primary, primary and lower secondary) or moderate/high (upper secondary and post-secondary) education.

### Analyses

Descriptive statistics were presented separately for men and women at all time points.

To assess the longitudinal association between family caregiving and depressive symptoms, mixed-models were performed with family caregiving as the main independent variable and depressive symptoms as the dependent variable, stratified by gender. Between- and within-person effects were estimated separately. The between-person part of the association compared depressive symptoms between workers with and without family caregiving tasks and was based on the individual mean values of the time varying independent variables ($Y_{it}=\beta_{0}+\sum_{j=1}^{J}\beta_{\mathrm{Bj}}{\bar{X}}_{ij}+\epsilon_{it}$) (28). The within-person part of the association provided insight into the changes in depressive symptoms when family caregiving changed (eg, when an individual started or stopped with caregiving tasks) and was based on the differences between the observations and the individual mean value ($Y_{it}=\beta_{0}+\sum_{j=1}^{J}\beta_{\mathrm{Wj}}\left(X_{\mathrm{ijt}}-{\bar{X}}_{\mathrm{ij}}\right)+\epsilon_{it}$) (28). The crude between- and within-person effects of family caregiving on depressive symptoms were calculated in separate univariable models. In separate multivariable models the between- and within-person effects were adjusted for age, partner status and education, since they might influence the association between family caregiving and depressive symptoms. Random intercepts were included in all models to adjust for the correlated observations within the respondents. The xtreg (be/fe) procedure in STATA V.15.1 was used for the analyses. Respondents with one observation only contributed to the between-person effects. Respondents with more than one observation contributed to both the between- and within-person effects. No additional missing data analysis was performed, since the percentage of missing data was <5% for all variables (29). Also, research showed that imputation of missing values is not necessary before performing a longitudinal mixed-model analyses, regardless of the missing data mechanism (30).

To test for gender differences, the crude and adjusted between- and within-person regression coefficients of family caregiving were tested by a linear contrast (31). Z-scores were computed by dividing the difference between coefficients by its standard error, which was defined as the square root of the sum of the variances of the two coefficients (*B*_female_–*B*_male_)/√(*SE*_female_^2^ + *SE*_male_^2^) (31). Z-scores > |1.96| indicated gender differences. A sensitivity analysis with interaction terms between gender and family care was performed in supplementary table S2.

Next, we explored the role of work characteristics in the longitudinal association between family care and depressive symptoms among older workers by adding them separately to the multivariable models. Because all analyses were stratified by gender, the role of work characteristics was assessed separately for men and women.

## Results

The characteristics of the sample are presented in table 1. Across the three measurements, women (32%) provided family care more often than men (20%). About 7% of women provided family care for one year, about 6% provided family care for two years and more than 19% provided family care in all three years. Men provided family care for one year in about 6% of the cases, about 4% provided family care in two years, and almost 11% provided family care in all three years. For respondents with observations in all three years, changes in family caregiving are presented in table 2. The presence of depressive symptoms was higher for women across the three measurements compared to men (5.8 versus 5). Across all measurements, men had higher scores for autonomy in work (3.8 versus 3.6) and mental workload (4.2 versus 4.1), and worked more hours per week on average (38.2 versus 27.3) compared to women. Women had higher scores for social support at work (3.6 versus 3.5, but only in 2016) and for emotional workload (2.5 versus 2.3) than men.

**Table 1:** Description of the sample. [SD=standard deviation; T=time.]

	Women (N=5797)						Men (N=6650)						Women-Men ^a^		
		
	2015 (T0)		2016 (T1)		2017 (T2)		2015 (T0)		2016 (T1)		2017 (T2)		2015 (T0)	2016 (T1)	2017 (T2)
	Mean (SD)	%	Mean (SD)	%	Mean (SD)	%	Mean (SD)	%	Mean (SD)	%	Mean (SD)	%			
Characteristics workers															
Family caregiving		31.6		32.9		32.7		18.1		20.4		21.3	***	***	***
Age (45–72)	53.2 (6.1)		54.3 (6.0)		54.8 (5.8)		54.7 (6.3)		55.6 (6.0)		56.2 (5.8)	***	***	***
Partner		71.9		71.5		71.9		80.4		79.8		79.9	***	***	***
Education ^b^		73.5		74.6		74.4		75.9		76.4		76.5	**		
Mental health															
Depressive symptoms (0–30)	5.8 (4.9)		5.8 (4.9)		5.8 (4.9)		5.0 (4.6)		4.9 (4.5)		5.1 (4.6)		***	***	***
Work characteristics															
Hours of work (1–96)	27.2 (10.6)		27.3 (10.6)		27.6 (10.4)		38.6 (9.5)		38.2 (9.6)		38.2 (9.7)		***	***	***
Social support at work (1–5)	3.6 (0.9)		3.6 (0.8)		3.6 (0.9)		3.5 (0.8)		3.5 (0.8)		3.5 (0.8)		*	
Autonomy (1–5)	3.6 (0.8)		3.6 (0.8)		3.6 (0.8)		3.8 (0.7)		3.8 (0.7)		3.8 (0.7)		***	***	***
Emotional workload (1–5)	2.5 (0.9)		2.5 (0.9)		2.5 (0.9)		2.3 (0.9)		2.3 (0.8)		2.3 (0.8)		***	***	***
Mental workload (1–5)	4.1 (0.7)		4.1 (0.8)		4.1 (0.7)		4.2 (0.7)		4.2 (0.6)		4.2 (0.6)		***	***	***

a Chi-square and t-tests.

b Education was only measured at baseline (baseline year differs between persons).

* P <0.05.

** P <0.01.

*** P <0.001.

**Table 2:** Changes in family care provision for respondents with observations in three measurement years (N=6521).

Caregiver in			Total	Men	Women
			
2015	2016	2017	N (%)	N (%)	N (%)
Yes	No	No	271 (4.2)	122 (3.5)	149 (5)
No	Yes	No	196 (3)	98 (2.8)	98 (3.3)
No	No	Yes	365 (5.6)	197 (5.6)	168 (5.6)
Yes	Yes	No	233 (3.6)	93 (2.6)	140 (4.7)
Yes	No	Yes	116 (1.8)	48 (1.4)	68 (2.3)
No	Yes	Yes	267 (4.1)	127 (3.6)	140 (4.7)
Yes	Yes	Yes	957 (14.7)	379 (10.7)	578 (19.4)

### Gender differences in depressive symptoms by combining paid work and family care

The models showed that family caregiving was positively associated with depressive symptoms between- and within persons for both women and men (table 3). The adjusted between-person effect showed that employed family caregivers experienced more depressive symptoms compared to non-caregiving employees. The effect sizes were similar for women and men (women B= 0.80, 95% CI 0.52–1.08; men B=0.75, 95% CI 0.45–1.05; Z-score 0.24). The adjusted within-person effect indicated that a change in family care was related to changes in depressive symptoms (eg, starting with family caregiving tasks was associated with an increase in depressive symptoms, while stopping caregiving was associated with reduced depressive symptoms). The effect sizes did not differ between women and men (women B=0.32, 95% CI 0.08–0.56; men B=0.25, 95% CI 0.01–0.48; Z-score 0.41).

**Table 3:** Mixed-model analysis family care on depressive symptoms (N women=13 125 observations from 5795 respondents; N men=15 272 observations from 6643 respondents). P-values <0.05 were considered statistically significant (in bold). The between-person and within-person effects were estimated in separate models. [BP=between-person effect; SE=standard error; WP=within-person effect]

	Women			Men			Z-score
	
	B	SE	95% CI	B	SE	95% CI	
Model 1							
BP family caregiving (vs. no caregiving)	**0.73**	0.14	0.46–1.00	**0.60**	0.15	0.31–0.88	0.63
WP family caregiving (vs. no caregiving)	**0.34**	0.12	0.10–0.58	**0.25**	0.12	0.02–0.48	0.53
Model 2							
BP family caregiving (vs. no caregiving)	**0.80**	0.14	0.52–1.08	**0.75**	0.15	0.45–1.05	0.24
WP family caregiving (vs. no caregiving)	**0.32**	0.12	0.08–0.56	**0.26**	0.12	0.01–0.48	0.41
BP age	**-0.06**	0.01	-0.08– -0.04	**-0.05**	0.01	-0.07– -0.04	-0.71
WP age	**0.10**	0.04	0.03–0.17	0.05	0.03	-0.01–0.11	1.00
BP partner (vs. no partner)	**-1.58 ^a^**	0.14	-1.85– -1.32	**-1.18 ^a^**	0.14	-1.45– -0.92	**-2.02**
WP partner (vs. no partner)	**-0.97**	0.33	-1.61– -0.32	-0.38	0.28	-0.92–0.16	-1.36
BP high education (vs. low education)	**-0.57**	0.14	-0.83– -0.30	**-0.86**	0.12	-1.10– -0.62	1.57

a Z-score was > |1.96| (indicating a significant difference between men and women).

### The role of work characteristics in the association between family care and depressive symptoms

Correcting for working hours did not influence the effect of family caregiving between- and within-persons for women. For men, the effect of family caregiving somewhat reduced on the within-person level (table 4, model 2). Working more hours was associated with fewer depressive symptoms for men on the between-person level, while an increase in working hours was related to an increase in depressive symptoms for men on the within-person level. For women, working hours was not associated with depressive symptoms. Adding autonomy in work to the model slightly reduced the effect of family caregiving within-persons for men (model 3). Higher levels of autonomy in work were associated with fewer depressive symptoms for women (between- and within-person) and men (between-person).

**Table 4:** The role of work characteristics in the association between family care and depressive symptoms (N women=13 125 observations from 5795 respondents / N men=15 272 observations from 6643 respondents), P-values <0.05 were considered statistically significant (in bold). All models were adjusted for age, partner status and education. The between-person and within-person effects were estimated in separate models. [BP=between-person effect; SE=standard error; WP=within-person effect].

	Women				Men				Z-score
	
	B	SE	95% CI	Δ ^a^(%)	B	SE	95% CI	Δ ^a^(%)	
Model 1									
BP family caregiving (vs. no caregiving)	**0.80**	0.14	0.52–1.08		**0.75**	0.15	0.45–1.05		0.24
WP family caregiving (vs. no caregiving)	**0.32**	0.12	0.08–0.56		**0.26**	0.12	0.01–0.48		0.35
Model 2									
BP damily caregiving (vs. no caregiving)	**0.80**	0.14	0.52–1.08	0	**0.74**	0.15	0.45–1.03	1	0.29
WP family caregiving (vs. no caregiving)	0.32	0.12	0.08–0.57	0	**0.24**	0.12	0.01–0.48	8	0.47
BP work hours	-0.03	0.01	-0.04–-0.02		**-0.03**	0.01	-0.04– -0.02		0.00
WP work hours	0.01	0.01	-0.02–0.02		**0.02**	0.01	0.01–0.04		-0.71
Model 3									
BP family caregiving (vs. no caregiving)	**0.76**	0.14	0.48–1.04	5	**0.71**	0.15	0.42–1.01	5	0.24
WP family caregiving (vs. no caregiving)	**0.32**	0.12	0.09–0.57	0	0.23	0.12	-0.01–0.46	12	0.53
BP autonomy	**-0.90**	0.08	-1.07–0.74		**-1.11**	0.08	-1.27– -0.96		1.86
WP autonomy	**-0.56**	0.09	-0.74–0.38		**-0.50**	0.08	-0.67– -0.34		-0.50
Model 4									
BP family caregiving (vs. no caregiving)	**0.72**	0.14	0.45–0.99	10	**0.64**	0.15	0.35–0.92	15	0.39
WP family caregiving (vs. no caregiving)	**0.31**	0.12	0.07–0.55	3	0.22	0.12	-0.02–0.45	15	0.53
BP social support	**-1.48**	0.07	-1.63–1.34		**-1.57**	0.07	-1.71– -1.43		0.91
WP social support	**-0.76**	0.07	-0.89–-0.62		**-0.70**	0.06	-0.82– -0.57		-0.65
Model 5									
BP family caregiving (vs. no caregiving)	**0.58**	0.14	0.30–0.85	28	**0.51**	0.14	0.23–0.79	32	0.35
WP family caregiving (vs. no caregiving)	**0.32**	0.12	0.08–0.56	0	**0.25**	0.12	0.02–0.48	4	0.41
BP emotional workload	**1.13 ^a^**	0.07	0.99–1.27		**1.59 ^a^**	0.07	1.46–1.72		**-4.65**
WP emotional workload	**0.74**	0.08	0.58–0.89		**0.84**	0.06	0.72–0.97		-1.00
Model 6									
BP family caregiving (vs. no caregiving)	**0.78**	0.14	0.50–1.06	3	**0.72**	0.15	0.42–1.01	4	0.29
WP family caregiving (vs. no caregiving)	**0.33**	0.12	0.08–0.57	3	**0.24**	0.12	0.01–0.48	8	0.53
BP mental workload	**0.35**	0.09	0.18–0.52		**0.29**	0.09	0.12–0.47		0.47
WP mental workload	0.01 ^b^	0.09	-0.17–0.19		**0.25 ^b^**	0.08	0.09–0.42		**-1.99**

a Δ = Change in coefficient family caregiving after correction for work characteristic.

b Z-score was > |1.96| (indicating a significant difference between men and women).

Social support at work (model 4) reduced the effect of family caregiving on depressive symptoms for women (between-person) and men (between- and within-person). This indicated that the unfavorable effect of family caregiving was partly compensated for by the favorable effect of social support. Receiving more social support at work was related to fewer depressive symptoms for women (between- and within-person) and men (between-person).

Emotional workload reduced the effect of family caregiving for women and men on the between-person level (model 5). This indicated that the effect of family caregiving on depressive symptoms was partly due to the unfavorable effect of emotional workload on depressive symptoms. Having higher levels of emotional workload were associated with more depressive symptoms for women and men on between-persons and within-persons. The Z-score (-4.65) indicated that the effect of emotional workload was stronger for men compared to women at the between-person level.

Mental workload slightly reduced the effect of family caregiving on depressive symptoms for men on the within-person level (model 6). Higher levels of mental workload were related to more depressive symptoms for women (between-persons) and men (between-persons and within-persons). The Z-score (-1.99) indicated that the effect of mental workload was stronger for men than for women.

## Discussion

This study showed that employed family caregivers experienced more depressive symptoms than non-caregiving employees, and beginning or ending family caregiving was associated with a change in depressive symptoms for both women and men. Social support at work and emotional workload reduced the effect of family care on depressive symptoms for women and men. Autonomy in work, mental workload and working hours only slightly reduced the association between family care and depressive symptoms.

### No clear gender differences in mental health when combining paid work and family care

The longitudinal association between family care and mental health was similar for male and female employees. This is in line with findings from a longitudinal study that linked family caregiving to worsened mental health for both men and women (32). On the contrary, the results differ from a meta-analysis that reported small gender differences in burden and depressive symptoms caused by family caregiving (6), and a cohort study that showed that men’s depressive state was less sensitive to family caregiving than that of women (7). However, these studies did not specifically focus on employees. Having occupational responsibilities next to caregiving tasks could be a double burden for both men and women (33). Conversely, a meta-review has shown that work can be beneficial for employees’ mental health (34), and spending time at work could offer respite from caregiving demands (35). Hence, it could be that there are less gender-related differences in mental health by family caregiving in the working population compared to a general population.

In line with prior findings, women provided family care more often than men (12), but when men did provide family care the effect of caregiving on mental health was comparable. On the one hand, it could be that gender differences are less pronounced within the group that already combines paid work and family care, but are particularly present in the decision to take on care responsibilities. This possibly decreases differences within the group of family caregivers and may explain why no differences in mental health between women and men were found.

On the other hand, it is possible that gender differences in mental health by family caregiving are decreasing due to changing gender roles (36). Pinquart and colleagues (6) argued that caregiving experiences of men and women have become more similar over the years. Also, about three quarter of the employed women and men in this study were highly educated. Hence, gender differences in mental health resulting from family caregiving may be limited among older workers in white-collar jobs. An alternative explanation could be that mental health is more strongly determined by care demands and the availability of additional caregivers than by gender differences (6). When interpreting the results, we should bear in mind that the effect sizes of family care on depressive symptoms among older employees were relatively small. However, a small average effect on population level could still mean that there are subgroups in which the effects are larger (eg, those involved in intensive care situations). Future research could assess the role of care characteristics (eg, intensity, illness trajectory, sharing care tasks with others) in addition to work characteristics to identify subgroups that experience mental health issues by combining family caregiving and work. Furthermore, it should be considered that the mean score of depressive symptoms was relatively low. Although women experienced slightly more depressive symptoms than men, the mean score was for both below the cut-off point of ten that is advised to identify clinically relevant depression (22, 37). This could be a ‘healthy worker effect’, ie, employees tend to have better health status compared to the general population (38). However, the results showed that family caregiving contributed to depressive symptoms. This indicates that family caregiving is a relevant issue for the mental health of older employees.

### The importance of work characteristics

In line with former research (4, 39), the results showed that social support at work could protect employees against depressive symptoms. Inclusion of social support reduced the effect of family caregiving in the between-person models, for both men and women, which may reflect that people who provide care are, compared to non-caregivers, more likely to have less social support. However, in contrast to our findings, Earle and colleagues (39) found that the protecting effects of work characteristics were somewhat larger for women. An explanation for this difference could be that the women in their sample worked about 39 hours per week on average, while women in the current study approximately worked 27 hours per week on average. Work characteristics such as social support possibly have a stronger protecting effect on mental health of women who combine family care with a fulltime job. From previous research (40), it is known that autonomy in work can also protect employees against depressive symptoms. However, in our study, autonomy in work only slightly reduced the association between family care and depressive symptoms. An explanation could be that, autonomy in the current study, reflects autonomy in work tasks (eg, deciding how to perform work tasks) rather than having control over work arrangements, such as the decision to come in late or leave early. The latter might be of more importance in accommodating work and family care responsibilities (41). Other work resources (eg, flexible work hours, social support) might be more important in explaining – and preventing – depressive symptoms among working family caregivers. The results indicated that emotional and mental workload were risk factors for adverse mental health. Only emotional workload played a role in the association between family care and depressive symptoms for both women and men. This suggests that people who provide care are, compared to non-caregivers, more likely to take on emotional workloads, whether at work or in caregiving. As employees who combine paid work and family care often have jobs with high emotional workload (42–44), they could benefit from support by employers to prevent mental health problems. Gender effects could play a role here, since jobs with high emotional workload (eg, healthcare) are often fulfilled by women or men with feminine traits (42, 45). Working hours only slightly influenced the association between family care and depressive symptoms. Although men worked more hours than women, differences in depressive symptoms between women and men with caregiving tasks were not found. In line with social role theory (46), an explanation could be that women generally work fewer hours per week but more often combine their paid work with other responsibilities, such as family care or volunteer work, while men usually work more hours but often are less engaged in other roles. Also, research has shown that hours spent in roles was more important in relation to mental health than the role combination in itself (47). Future research could also include the intensity of caregiving and time spent in other societal roles fulfilled by employed women and men in relation to mental health.

### Methodological considerations

The strength of the longitudinal design is that information about family caregiving and mental health was available for a large number of employees in subsequent years of the prospective cohort, which made it possible to assess changes over time. Another strong point is that the between- and within-person effects were estimated separately, which enabled comparison of depressive symptoms between caregivers and non-caregivers, as well as determine changes in depressive symptoms when someone took up or ended caregiving tasks. The results showed that the between-person effects were often larger than the within-person effects. This confirms previous findings that the magnitude of effects could differ between- and within-persons (17, 48). Work characteristics were not specifically measured in the context of family caregiving. Social support at work mainly included support in work-related problems and not problems related to the combination of work and family care per se. Although we could argue that employees who receive support in work-related problems are likely to also receive support in other types of problems, the role of social support at work might be underestimated. Future studies could specifically include work characteristics (eg, social support, autonomy in work) in the context of family caregiving.

### Practical implications

Our findings indicated that family caregiving was positively associated with depressive symptoms over time, underlining the need to prevent mental health problems. One-fifth of male employees and one-third of female employees combined work with family care, and these numbers are likely to increase in the coming years. Hence, older workers with family caregiving tasks could benefit from more social support in work. To realize this, more attention is needed for employees with caregiving tasks from employers, supervisors and colleagues, in which open communication about family caregiving is important. In addition, supervisors can be better informed by their organization about support options for family caregivers (49).

### Concluding remarks

Caregiving demands can impact the mental health of older employees in a negative way. Although women provide family care more often than men, the longitudinal association between family care and mental health was similar for male and female employees. Resources at work (ie, social support) could protect older employees who give family care against depressive symptoms. Employed family caregivers who have jobs with high emotional workload could in particular benefit from support by employers to prevent mental health problems. This study underlines the need for support from employers to prevent mental health problems among employees with caregiving tasks. More research is needed regarding the relative impact of the care context compared to the work context of working family caregivers.

### Ethics approval

The Medical Ethical Committee of the VU University Medical Center (Amsterdam, the Netherlands) declared that the Medical Research Involving Human Subjects Act does not apply to the STREAM study and indicated to have no objection to the execution of this research (reference number 2012/080). The TNO Institutional Review Board has expressed a positive recommendation based on the research design, privacy and ethical aspects and the burden and the risks to the research participants (reference number 2019-100).

### Funding

The ZonMw Gender and Health programme funded this work (grant number 80-84900-98-321). ZonMw was not involved in the analysis or writing of this paper.

## Supplementary material

Supplementary material

## References

[1] Rijs KJ, Cozijnsen R, Deeg DJ (2012). The effect of retirement and age at retirement on self-perceived health after three years of follow-up in Dutch 55–64-year-olds. Ageing Soc.

[2] Josten E, de Boer A (2015). Concurrentie tussen mantelzorg en betaald werk. [Competition between informal care and paid work] Den Haag:Sociaal en Cultureel Planbureau.

[3] Joling KJ, O'Dwyer ST, Hertogh CM, van Hout HP (2018). The occurrence and persistence of thoughts of suicide, self-harm and death in family caregivers of people with dementia:a longitudinal data analysis over 2 years. Int J Geriatr Psychiatry.

[4] Broese Van Groenou M, Schakel S, Tolkacheva N (2015). Werk en mantelzorg. Een risico voor de psychische gezondheid?. [Work and care. A risk to mental health?] TvA.

[5] Farfan-Portet MI, Popham F, Mitchell R, Swine C, Lorant V (2010). Caring, employment and health among adults of working age:evidence from Britain and Belgium. Eur J Public Health.

[6] Pinquart M, Sörensen S (2006). Gender differences in caregiver stressors, social resources, and health:an updated meta-analysis. J Gerontol B Psychol Sci Soc Sci.

[7] Wakabayashi M, Kureishi W (2018). Differences in the effects of informal family caregiving on health and life satisfaction between wives and husbands as caregivers. Rev Dev Econ.

[8] Lee Y, Tang F (2015). More caregiving, less working:caregiving roles and gender difference. J Appl Gerontol.

[9] Van Echteld P, Croezen S, Vlasblom J, De Voogd-Hamelink M, Mattijssen L (2016). Aanbod van Arbeid 2016. Werken, zorgen en leren op een flexibele arbeidsmarkt. [Supply of labour 2016. Working, caring and learning in a flexible labour market].

[10] Gaugler JE, Given WC, Linder J, Kataria R, Tucker G, Regine WF (2008). Work, gender, and stress in family cancer caregiving. Support Care Cancer.

[11] Haberkern K, Schmid T, Szydlik M (2015). Gender differences in intergenerational care in European welfare states. Ageing Soc.

[12] de Boer A, Plaisier I, de Klerk M (2019). Werk en Mantelzorg. Kwaliteit van leven en het gebruik van ondersteuning op werk. [Work and informal care. Quality of life and the use of support at work].

[13] CBS Psycho-sociale arbeidsbelasting werknemers;beroep.

[14] CBS Psychosociale arbeidsbelasting (PSA) werknemers;geslacht en leeftijd.

[15] Schaufeli W, Bakker A (2013). De psychologie van arbeid en gezondheid. [The psychology of work and health].

[16] Blanch A, Aluja A (2012). Social support (family and supervisor), work–family conflict, and burnout:sex differences. Hum Relat.

[17] Twisk JW, de Vente W (2019). Hybrid models were found to be very elegant to disentangle longitudinal within- and between-subject relationships. J Clin Epidemiol.

[18] Ybema JF, Geuskens GA, van den Heuvel SG, de Wind A, Leijten FR, Joling CI (2014). Study on Transitions in Employment, Ability and Motivation (STREAM):the design of a four-year longitudinal cohort study among 15,118 persons aged 45 to 64 years. Br J Med Med Res.

[19] Van den Heuvel SG, Geuskens GA, Bouwhuis S, Niks I (2018). Study on transitions in employment, ability and motivation (STREAM):methodologisch rapport (2010-2017). Leiden.

[20] Van den Hoofdakker R, Albersnagel F, De Cuyper H, Vandereycken W, Hoogduin C.A.L, Emmelkamp P. M. G (1994). Stemmingsstoornissen. Handboek Psychopathologie deel 1.

[21] Radloff L (1977). The CES-D Scale:A self-report depression scale for research in the general population. Appl Psychol Meas.

[22] Andresen EM, Malmgren JA, Carter WB, Patrick DL (1994). Screening for depression in well older adults:evaluation of a short form of the CES-D (Center for Epidemiologic Studies Depression Scale). Am J Prev Med.

[23] Stall NM, Campbell A, Reddy M, Rochon PA (2019). Words matter:the language of family caregiving. J Am Geriatr Soc.

[24] Karasek R (1985). Job Content Questionnaire and user's guide.

[25] Karasek R, Brisson C, Kawakami N, Houtman I, Bongers P, Amick B (1998). The Job Content Questionnaire (JCQ):an instrument for internationally comparative assessments of psychosocial job characteristics. J Occup Health Psychol.

[26] Kristensen TS, Hannerz H, Høgh A, Borg V (2005). The Copenhagen Psychosocial Questionnaire--a tool for the assessment and improvement of the psychosocial work environment. Scand J Work Environ Health.

[27] Koppes L, De Vroome E, Mol M, Janssen B, Van den Bossche S (2010). The Netherlands working conditions survey 2007:methodology and overall results [Nationale Enquête Arbeidsomstandigheden 2009. Methodologie en globale resultaten].

[28] Twisk J (2019). Applied Mixed Model Analysis:a practical guide.

[29] Schafer JL (1999). Multiple imputation:a primer. Stat Methods Med Res.

[30] Twisk J, de Boer M, de Vente W, Heymans M (2013). Multiple imputation of missing values was not necessary before performing a longitudinal mixed-model analysis. J Clin Epidemiol.

[31] Paternoster R, Brame R, Mazerolle P, Piquero A (1998). Using the correct statistical test for the equality of regression coefficients. Criminology.

[32] Hajek A, König HH (2016). Informal caregiving and subjective well-being:evidence of a population-based longitudinal study of older adults in Germany. J Am Med Dir Assoc.

[33] Schmitz H, Stroka MA (2013). Health and the double burden of full-time work and informal care provision—evidence from administrative data. Labour Econ.

[34] Modini M, Joyce S, Mykletun A, Christensen H, Bryant RA, Mitchell PB (2016). The mental health benefits of employment:results of a systematic meta-review. Australas Psychiatry.

[35] Joseph G, Joseph A (2019). Exploring employment as a space of respite and resistance for family caregivers. Health Soc Care Community.

[36] Comas-d'Argemir D, Soronellas M (2019). Men as carers in long-term caring:doing gender and doing Kinship. J Fam Issues.

[37] Boey KW (1999). Cross-validation of a short form of the CES-D in Chinese elderly. Int J Geriatr Psychiatry.

[38] Chowdhury R, Shah D, Payal AR (2017). Healthy worker effect phenomenon:revisited with emphasis on statistical methods–a review. Indian J Occup Environ Med.

[39] Earle A, Heymann J (2011). Protecting the health of employees caring for family members with special health care needs. Soc Sci Med.

[40] Wheatley D (2017). Autonomy in paid work and employee subjective well-being. Work Occup.

[41] Arksey H (2002). Combining informal care and work:supporting carers in the workplace. Health Soc Care Community.

[42] DePasquale N, Davis KD, Zarit SH, Moen P, Hammer LB, Almeida DM (2016). Combining formal and informal caregiving roles:the psychosocial implications of double-and triple-duty care. J Gerontol B Psychol Sci Soc Sci.

[43] Häusler N, Bopp M, Hämmig O (2017). Informal caregiving, work-privacy conflict and burnout among health professionals in Switzerland - a cross-sectional study. Swiss Med Wkly.

[44] Boumans NP, Dorant E (2014). Double-duty caregivers:healthcare professionals juggling employment and informal caregiving. A survey on personal health and work experiences. J Adv Nurs.

[45] O'Connor T (2015). Men choosing nursing:negotiating a masculine identity in a feminine world. J Men's Stud.

[46] Eagly AH, Wood W (2016). Social role theory of sex differences. The Wiley Blackwell encyclopedia of gender and sexuality studies.

[47] Bijnsdorp F, Suanet B, Broese van Groenou M (2018). Het combineren van meerdere rollen onder ouderen:vermindert of verbetert dit het welbevinden?[Combining multiple roles among the elderly:does this reduce or enhance well-being?]. Mens Maatschappij.

[48] Hoffman L, Stawski RS (2009). Persons as contexts:evaluating between- and within-person effects in longitudinal analysis. Res Hum Dev.

[49] Plaisier I, Broese van Groenou MI, Keuzenkamp S (2015). Combining work and informal care:the importance of caring organisations. Hum Resour Manage J.

