# Supplementary material for: Associations of combining paid work and family care with gender-specific differences in depressive symptoms among older workers and the role of work characteristics
Source: Scand J Work Environ Health. 2022 Mar 31;48(3):190–9. doi: 10.5271/sjweh.4014 (PMC9523461; doi:10.5271/sjweh.4014)
Supplement: Supplementary material [file SJWEH-48-190-S001.pdf]

Associations of combining paid work and family care with gender-specific differences in depressive symptoms among older workers and the role of work characteristics[1]

by Femmy M Bijnsdorp, MSc,[2] Allard J van der Beek, PhD, Marjolein I Broese van Groenou, PhD, Karin I Proper, PhD,
Swenneke G van den Heuvel, PhD, Cécile RL Boot, PhD

1. *Supplementary material*
2. *Correspondence to: Femmy M. Bijnsdorp, Department of Public and Occupational Health, Amsterdam UMC, P.O. Box 7057, 1007 MB Amsterdam, The Netherlands. [E-mail: f.bijnsdorp@amsterdamumc.nl]*

Table S1. Between-person and within-person variance of depressive symptoms and work characteristics

	Between-person variance	Within-person variance
Depressive symptoms	22.44	17.12
Hours of work	0.25	0.08
Social support at work	0.66	0.49
Autonomy	0.53	0.29
Emotional workload	0.74	0.43
Mental workload	0.48	0.29

Table S2. Sensitivity analysis interaction between family care and gender on depressive symptoms (n = 28,397 observations from 12,438 respondents)

	B	se	95% CI
**Model 1**			
BP Family caregiving (vs. no caregiving)	**0.60**	0.15	0.30 – 0.89
WP Family caregiving (vs. no caregiving)	**0.25**	0.12	0.01 – 0.49
BP Women (vs. men)	**0.71**	0.09	0.53 - 0.90
BP Family caregiving x Women	0.13	0.20	-0.26 - 0.53
WP Family caregiving x Women	0.09	0.17	-0.24 - 0.42
**Model 2**			
BP Family caregiving (vs. no caregiving)	**0.75**	0.15	0.44 – 1.05

WP Family caregiving (vs. no caregiving)	0.24	0.12	-0.00 – 0.58
BP Women (vs. men)	**0.48**	0.10	0.29 - 0.67
BP Family caregiving x Women	0.05	0.21	-0.35 - 0.46
WP Family caregiving x Women	0.09	0.17	-0.25 - 0.42
BP Age	**-0.05**	0.01	-0.07 – -0.04
WP Age	**0.07**	0.02	0.03 – 0.12
BP Partner (vs. no partner)	**-1.39**	0.10	-1.58 – -1.21
WP Partner (vs. no partner)	**-0.64**	0.21	-1.06 – -0.23
BP High education (vs. low education)	**-0.71**	0.09	-0.89 – -0.53

Notes: BP = Between-person effect; WP = Within-person effect. The between-person and within-person effects were estimated in separate models.
P-values < 0.05 were considered statistically significant (in bold).

The main effects of gender and education were omitted from the within-person analysis because there was no within-person variation.
